# Evaluation of a National Health Service Machine-Learning Model for Hypertension Case-Finding: Retrospective Cohort Study

**DOI:** 10.2196/87084

**Published:** 2026-09-15

**Authors:** Gloria Ihenetu, Ahmad Alkhatib, Vesselin Novov, Thomas Beaney, Azeem Majeed, Paul Aylin, Thomas Woodcock

**Affiliations:** 1 Department of Primary Care and Public Health, Imperial College London, White City Campus, 90 Wood Lane, London, W12 0BZ, United Kingdom, 44 020 7594 5863

**Keywords:** hypertension, case-finding, machine learning, general practice, health equity

## Abstract

**Background:**

Hypertension is a leading preventable cause of cardiovascular disease, yet a substantial proportion of adults remain undiagnosed, limiting opportunities for early intervention. A predictive model was commissioned by the North West London (NWL) Integrated Care Board to identify undiagnosed hypertension. The model was developed using health records from the Whole Systems Integrated Care (WSIC) database.

**Objective:**

We aimed to independently evaluate the predictive performance of the model as it would be encountered in deployment, how performance varied by demographic characteristics, and practical utility.

**Methods:**

To evaluate the predictive model, we conducted a retrospective cohort study of 1,802,920 individuals aged 16 years or older, registered with a general practice in NWL, and with no prior diagnosis of hypertension from May 2023 to May 2024. We assessed the model’s predictions against recorded hypertension status using medical diagnoses and blood pressure records. Logistic regression models were used to assess the sensitivity and specificity of the model’s predictions by sociodemographic groups. We also compared the model’s performance against a more interpretable regression approach.

**Results:**

The model yielded an overall sensitivity of 62.7% (95% CI 62.5-62.8) and specificity of 60.7% (95% CI 60.5-60.8). Positive predictive value ranged from 31.5% (95% CI 31.2-31.8) to 42.9% (95% CI 42.5-43.2), and negative predictive value ranged from 77.6% (95% CI 77.2-77.9) to 84.9% (95% CI 84.7-85.2). Sensitivity was higher in older adults and Black patients; specificity was higher in younger adults, female patients, and White patients. Overall, sensitivity was higher for those living in areas of higher socioeconomic deprivation, while specificity was lower. These effects plateaued in the 2 least deprived quintiles of deprivation, which were comparable in both sensitivity and specificity. Predictions varied by age, with 96.2% (58,951/61,281) of those aged 70 to 79 predicted to have hypertension, whereas 0.08% of those aged 20 to 39 were predicted to have the condition. The model’s performance was comparable with a more interpretable logistic regression model.

**Conclusions:**

Despite the model’s relatively good performance for those without hypertension, the positive predictive value was low, and a significant proportion of true cases remained undetected. Furthermore, there was considerable variation in performance associated with demographic characteristics, suggesting tailored approaches to case-finding may be beneficial in ensuring equity across demographic groups. Especially given the importance of understanding possible biases in predictive models, we recommend that, where there is no loss in performance, more parsimonious, transparent models be selected for prediction in health care settings. The findings of this evaluation can guide the practical application of the model, inform enhancements, direct targeted screening initiatives, and support cost-benefit analyses for broader implementation to improve hypertension management.

## Introduction

Hypertension, or high blood pressure (BP), is a global public health issue that contributes to adverse health outcomes and to significant challenges for health systems [1,2]. It is the most common cardiovascular disease worldwide and increases the risk of major cardiovascular events by around 55%, cardiovascular mortality by 44%, and stroke by around 46% [2]. In the United Kingdom, hypertension is defined as a BP of ≥140/90 mm Hg with confirmation using home or ambulatory BP monitoring as recommended by the National Institute for Health and Care Excellence (NICE) [3]. In 2019, the estimated age-standardized prevalence of hypertension in the global adult population was 32% for females and 34% for males [3]. However, the diagnosed prevalence of hypertension is much lower because 3 in 10 adults estimated to have hypertension remain undiagnosed [4]. For example, in England, general practice (GP) records show that only around 16% of adults are diagnosed with hypertension, and Public Health England (PHE) estimates show that over 5 million people remain undiagnosed [5,6]. Furthermore, in North West London (NWL), more than 83,000 people out of the over 2 million residents in the area were estimated to have undiagnosed hypertension in 2023 [7,8]. Hypertension is also responsible for 12% of doctors’ appointments in England and an estimated annual cost of over £2 billion (GBP £1=US $1.35 as of September 01, 2026) [1].

Early detection, followed by interventions such as drugs and lifestyle changes, is a crucial evidence-based method for addressing hypertension and mitigating the associated health risks [9]. Thus, primary care teams worldwide have dedicated resources to detecting hypertension [10,11]. In recent years, machine-learning models have emerged as powerful tools to predict the incidence of diseases such as hypertension sooner [12-16]. As part of the 2023 NWL Health and Care Strategy, the NWL integrated care board commissioned a consultancy firm to develop a predictive model to aid in identifying the population at high risk of undiagnosed hypertension and to reduce health inequalities [7,8,17]. The consultancy firm developed a random forest model, trained using the NWL Whole Systems Integrated Care (WSIC) database containing electronic health records (EHRs) data for over 2.3 million individuals registered with a GP in NWL [18]. Census data at the Lower-layer Super Output Area level was also incorporated into the model to improve predictive accuracy, covering several social determinants of health. Medications and common comorbidities such as chronic obstructive pulmonary disease, asthma, diabetes, nondiabetic hyperglycemia, depression, anxiety, and chronic kidney disease were considered in the development of the model but were ultimately left out to avoid biasing the model toward patients who tend to have more interaction with the health system.

The model was developed using a train-test approach, specifically a 5-fold cross-validation, and a hold-out test set was used after training to evaluate the model to infer the validity of the model (Table 1). The model was deployed in May 2023.

**Table 1.** Overview of a predictive case-finding model to identify those at risk of undiagnosed hypertension within a population of individuals registered with a general practice in North West London in 2023.

Characteristic	Description
Model overview	
Type of algorithm	Random forest
Purpose	To identify those at high risk of undiagnosed hypertension
Data Source	
Source	Whole Systems Integrated Care and census data
Sample size	N=1,802,920
Final features used in model	Sociodemographic data and census data
Missing data	In extracting the training data, the model developers excluded a small number of patients whose gender was unknown or not recorded, or for whom data on presence or absence of long-term conditions was missing. The evaluation team used these same exclusion criteria applied to the evaluation dataset. There were a number of patients who had missing data for Index of Multiple Deprivation (IMD). Religion, language, and ethnicity variables included an explicit “unknown” or “not recorded” level. Other variables did not have missing data.
Model development	
Training or test split	5-fold cross-validation and hold-out test for evaluation
Model output	
Probability threshold	A 50% threshold was used for the general assessment of the model, but 60% was chosen as the prediction threshold. Equal to or above the selected threshold indicated a positive prediction status, while below it indicated a negative status
Risk scores	Model assigns risk score between 0 and 1 to each patient indicating risk of being diagnosed with hypertension
Evaluation team access	
Data	During the study, the evaluation team accessed predictions made by the model for eligible patients on May 19, 2023. While in principle the team could have accessed the training data used in developing the model, this was not needed to address the study aim
Model object(s)	The evaluation team did not have access to any model objects produced in training the model
Code	The evaluation team had access to the Python scripts used to clean data, train, and deploy the model

Following the deployment of the predictive model, the National Institute for Health Research (NIHR) Applied Research Collaboration (ARC) NWL team conducted an independent pragmatic end-user evaluation of the model’s performance using EHR data recorded after deployment.

Several similar models have been developed worldwide [12-15,19]. However, evaluations of these models were often conducted in controlled or development-phase environments, and few were clinically validated and evaluated using real-world data [20]. Furthermore, most evaluations assume that predictive performance metrics are similar across population groups, potentially amplifying biases inherent in the social determinants of health and access to health care, especially in the context of detecting undiagnosed conditions [13-15,21,22]. Lastly, many case-finding models were not evaluated using metrics that took the prevalence of the studied condition into account [12].

Our study is a real-world, pragmatic evaluation of the model’s performance 1 year after deployment. The aim was to evaluate the predictive performance of the model, how this performance varied across demographics, and what this means for the use of the model in practice.

## Methods

### Study Design and Data Source

We used a retrospective cohort study design with a study period from May 19, 2023, to May 19, 2024. Data were extracted on June 7, 2024. We used the WSIC database, which contains deidentified primary, secondary, tertiary, emergency department, and social care data for over 2.3 million individuals registered with a GP in NWL in 2023 [18].

### Study population

On May 19, 2023, the model generated hypertension predictions for 1,802,920 individuals, which we accessed as a static dataset. Many of these people would have been included in the original training cohort, given that they had met the inclusion criteria. This was a subset of the patient population in NWL who, at the time, were registered with a GP in NWL, were 16 years or older, and were not already diagnosed with hypertension. Patients with a recorded diagnosis of hypertension and an absent diagnosis date were excluded, as were patients with missing data on gender or prior long-term conditions. Since the predictive model only included male and female genders, we also excluded patients with recorded gender “other.” Full details of how we arrived at the full analytic sample are shown in the *Results* section.

We extracted 1 subsequent year of this cohort’s primary care medical record history on June 7, 2024, and used these data to evaluate the predictions made by the model. To ascertain each patient’s hypertension status over the study period, we defined a positive diagnosis of hypertension as a patient documented within the Quality and Outcomes Framework (QOF) hypertension register in the WSIC database [23]. Patients were considered to have a negative diagnosis if they had at least one recorded BP reading within the study period but no positive diagnosis recorded by their GP. This captures cases in which an initial BP reading was at least 140/90 mm Hg, but subsequent clinical investigation indicated that the patient did not have hypertension. This is in line with NICE guidelines [24]. If there was no interaction in which a BP measurement was taken and no hypertension diagnosis status documented, we counted the patient’s hypertension status as “not ascertained.” In our main analysis, we excluded individuals whose hypertension status was not ascertained.

We evaluated the model’s predictions against each patient’s known hypertension status, either positive or negative, derived from their GP records as above.

### Statistical Analyses

In the development of the model, sensitivity and specificity were computed using 2 probability thresholds, 50% (0.5) and 60% (0.6), with those over the threshold predicted to have hypertension. Specificity and overall performance of the model were best using the 60% threshold. Since the 60% threshold was selected by the consultancy firm for the prediction model, we primarily use it to report the results of the evaluation. Results using the 50% threshold are included in Table S1 in Multimedia Appendix 1.

Our primary method to evaluate the model was computing the sensitivity and specificity of the model-predicted hypertension status against the study-observed hypertension status. Since these statistics are independent of the prevalence, they are unaffected by differences in rates of ascertainment between those with hypertension and those without. We then computed the positive and negative predictive values (NPVs) and their respective CI from the observed sensitivity and specificity, using published national estimates of true hypertension prevalence [25]. We chose this approach because the premise of the model is that there are many cases of undiagnosed hypertension in the population, so labeling all who did not get a positive diagnosis during the study as not having hypertension would introduce significant misclassification bias into the evaluation. We conducted a descriptive analysis of the study population by sociodemographic groups, including gender, age, ethnicity, religion, language, deprivation, and residence in care homes. Using a confusion matrix, which summarizes the performance of predictive algorithms, we assessed the sensitivity and specificity of the model in the population overall [26]. We fitted multivariable logistic regression models to assess variation in sensitivity and specificity of predictions across demographic groups. Patients with missing data on ethnicity or Index of Multiple Deprivation (IMD) quintile were excluded from these regression models.

To understand the extent to which the use of a random forest model conveyed advantages in predictive power over simpler and more transparent regression models, we also fitted 2 logistic regression models, the first including age group, gender, ethnicity, and IMD quintile as predictors, and the second including only age group. We compared the sensitivity and specificity of these models to the random forest model. Further details are provided in Multimedia Appendix 1.

Finally, we conducted 2 sensitivity analyses. First, we reran our analysis while excluding those who died or deregistered from their GP during the study period and who were included in the main analysis. Second, we reran our analysis, classifying all those who did not have a positive diagnosis as not having hypertension. Results of both sensitivity analyses are presented in Tables S2-S8 in Multimedia Appendix 1.

### Ethical Considerations

The National Health Service (NHS) Health Research Authority West Midlands—Solihull Research Ethics Committee granted ethical approval covering this study under the WSIC Platform (REC: 23/WM/0196, IRAS: 333128), waiving the need for informed consent or compensation since the study was retrospective and no identifiable patient data were made available for or used in this research. Data access for this study was approved by the NWL Data Access Committee (ID-271‐2). All methods in this study were carried out in accordance with the guidelines and regulations for ethics and study approval.

## Results

**Figure 1.**
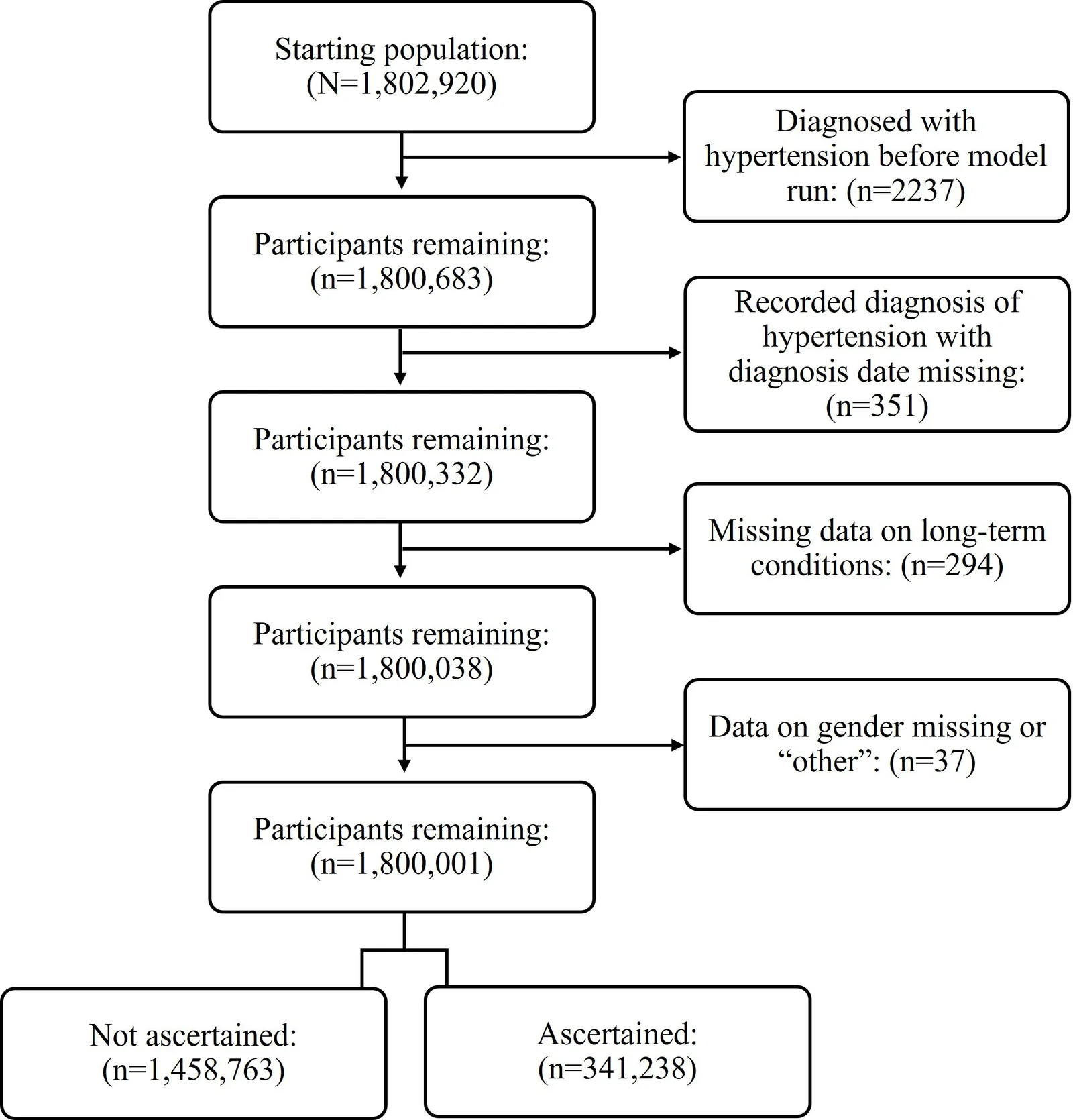
Flow diagram for the study sample.

Predictions were made for 1,802,920 patients, of whom 2919 did not meet the inclusion criteria (Figure 1). Of the excluded patients, 2237 had a diagnosis of hypertension with a date before the model was run. For 351, the hypertension diagnosis date was missing. A total of 294 patients were excluded because data on their long-term condition status were missing. A total of 37 patients had missing data on gender or were classified as “other.” The final analytical sample was 1,800,001, of whom 52.3% (n=942,068) were male (Table 2). The largest age group was 30 to 39 years, constituting 27.0% (n=485,253). Information about religion was unknown for most of the study population (n=1,378,233, 76.6%), while the majority ethnicity was White (n=806,999, 44.8%). The percentage of people whose hypertension status was ascertained during the study period increased with age, was lower in males than in females, and was higher for people living in areas of highest socioeconomic deprivation compared with the lowest.

Figure 1 shows the flow diagram of the study population. Inclusion and exclusion criteria for patients from the WSIC database.

**Table 2.** Descriptive statistics of the study population and ascertainment numbers and rates: patients registered with a North West London general practice, aged 16 or older, with no prior diagnosis of hypertension as of May 19, 2023.

Group	Total count, n (%)	Ascertained count, n (%)	Not ascertained count, n (%)
Total	1,800,001 (100)	341,238 (19.0)	1,458,763 (81.0)
Age (y)			
16‐19	76,298 (4.2)	6379 (8.4)	69,919 (91.6)
20‐29	396,322 (22.0)	41,527 (10.5)	354,795 (89.5)
30‐39	485,253 (27.0)	58,588 (12.1)	426,665 (87.9)
40‐49	370,776 (20.6)	74,166 (20.0)	296,610 (80.0)
50‐59	246,836 (13.7)	72,232 (29.3)	174,604 (70.7)
60‐69	137,825 (7.7)	50,338 (36.5)	87,487 (63.5)
70‐79	61,281 (3.4)	26,113 (42.6)	35,168 (57.4)
≥80	25,410 (1.4)	11,895 (46.8)	13,515 (53.2)
Gender			
Female	857,933 (47.7)	205,606 (24.0)	652,327 (76.0)
Male	942,068 (52.3)	135,632 (14.4)	806,436 (85.6)
Religion			
Declared	353,970 (19.7)	75,994 (21.5)	277,976 (78.5)
Atheist	59,835 (3.3)	10,310 (17.2)	49,525 (82.8)
Refused	7963 (0.4)	1470 (18.5)	6493 (81.5)
Unknown	1,378,233 (76.6)	253,464 (18.4)	1,124,769 (81.6)
Language			
Declared	479,119 (26.6)	94,045 (19.6)	385,074 (80.4)
English	654,928 (36.4)	143,126 (21.9)	511,802 (78.1)
Not recorded or refused	665,954 (37.0)	104,067 (15.6)	561,887 (84.4)
Ethnicity			
Asian or Asian British	482,721 (26.8)	99,093 (20.5)	383,628 (79.5)
Black or Black British	137,512 (7.6)	31,607 (23.0)	105,905 (77.0)
Other ethnic groups	234,894 (13.1)	38,872 (16.5)	196,022 (83.5)
Mixed	60,486 (3.4)	11,885 (19.6)	48,601 (80.4)
White	806,999 (44.8)	156,794 (19.4)	650,205 (80.6)
Missing	77,389 (4.3)	2987 (3.9)	74,402 (96.1)
IMD^a^ decile			
1 (most deprived)	37,839 (2.1)	8588 (22.7)	29,251 (77.3)
2	176,549 (9.8)	39,878 (22.6)	136,671 (77.4)
3	254,584 (14.1)	50,517 (19.8)	204,067 (80.2)
4	303,182 (16.8)	58,425 (19.3)	244,757 (80.7)
5	280,249 (15.6)	50,164 (17.9)	230,085 (82.1)
6	241,024 (13.4)	45,841 (19.0)	195,183 (81.0)
7	175,328 (9.7)	31,836 (18.2)	143,492 (81.8)
8	173,874 (9.7)	30,194 (17.4)	143,680 (82.6)
9	109,483 (6.1)	18,483 (16.9)	91,000 (83.1)
10 (least deprived)	43,971 (2.4)	6610 (15.0)	37,361 (85.0)
Missing	3918 (0.2)	702 (17.9)	3216 (82.1)
Care home resident	3253 (0.2)	1733 (53.3)	1520 (46.7)

a IMD: Index of Multiple Deprivation.

Observed incidence of hypertension over the study period increased with age from 0.2% (14/6379) in the 16 to 19 age group to 7.8% (2038/26113) in the 70 to 79 age group, with the oldest age group, 80 years or older, having a slightly lower incidence at 6.4% (758/11,895; Table 3). The 70 to 79 age group had the highest percentage of people predicted by the model to have undiagnosed hypertension, which was 96.2% (58,951/61,281). The 30 to 39 age group had the lowest percentage of predicted hypertension status (17/485,253, 0.004%), which is much lower than the observed percentage of hypertension diagnoses in this age group (1288/58,588, 2.2%).

**Table 3.** Counts and percentages of the study population with observed and predicted hypertension status by sociodemographic group: patients registered with a North West London general practice, aged 16 years or older, with no prior diagnosis of hypertension as of May 19, 2023.

Group	Predicted positive, n (%)	Predicted negative, n (%)	Observed positive, n (%)^a^	Observed negative, n (%)^a^
Total	402,298 (22.3)	1,397,703 (77.7)	16,061 (4.7)	325,177 (95.3)
Age (y)				
16‐19	2139 (2.8)	74,159 (97.2)	14 (0.2)	6365 (99.8)
20‐29	663 (0.2)	395,659 (99.8)	244 (0.6)	41,283 (99.4)
30‐39	17 (0.004)	485,236 (100.0)	1288 (2.2)	57,300 (97.8)
40‐49	61,456 (16.6)	309,320 (83.4)	3370 (4.5)	70,796 (95.5)
50‐59	137,553 (55.7)	109,283 (44.3)	4620 (6.4)	67,612 (93.6)
60‐69	119,978 (87.0)	17,847 (13.0)	3729 (7.4)	46,609 (92.6)
70‐79	58,951 (96.2)	2330 (3.8)	2038 (7.8)	24,075 (92.2)
≥80	21,541 (84.8)	3869 (15.2)	758 (6.4)	11,137 (93.6)
Gender				
Female	203,308 (23.7)	654,625 (76.3)	7668 (3.7)	197,938 (96.3)
Male	198,990 (21.1)	743,078 (78.9)	8393 (6.2)	127,239 (93.8)
Religion				
Declared	82,368 (23.3)	271,602 (76.7)	3429 (4.5)	72,565 (95.5)
Atheist	7560 (12.6)	52,275 (87.4)	331 (3.2)	9979 (96.8)
Refused	891 (11.2)	7072 (88.8)	37 (2.5)	1433 (97.5)
Unknown	311,479 (22.6)	1,066,754 (77.4)	12,264 (4.8)	241,200 (95.2)
Language				
Declared	115,462 (24.1)	363,657 (75.9)	4454 (4.7)	89,591(95.3)
English	156,055 (23.8)	498,873 (76.2)	6103 (4.3)	137,023 (95.7)
Not recorded or refused	130,781 (19.6)	535,173 (80.4)	5504 (5.3)	98,563 (94.7)
Ethnicity				
Asian or Asian British	135,882 (28.1)	346,839 (71.9)	5170 (5.2)	93,923 (94.8)
Black or Black British	52,805 (38.4)	84,707 (61.6)	1823 (5.8)	29,784 (94.2)
Other ethnic groups	35,545 (15.1)	199,349 (84.9)	1505 (3.9)	37,367 (96.1)
Mixed	9271 (15.3)	51,215 (84.7)	449 (3.8)	11,436 (96.2)
White	162,661 (20.2)	644,338 (79.8)	7006 (4.5)	149,788 (95.5)
Missing	6134 (7.9)	71,255 (92.1)	108 (3.6)	2879 (96.4)
IMD^b^ decile				
1 (most deprived)	12,250 (32.4)	25,589 (67.6)	444 (5.2)	8144 (94.8)
2	47,844 (27.1)	128,705 (72.9)	1716 (4.3)	38,162 (95.7)
3	60,603 (23.8)	193,981 (76.2)	2532 (5.0)	47,985 (95.0)
4	67,655 (22.3)	235,527 (77.7)	2600 (4.5)	55,825 (95.5)
5	61,365 (21.9)	218,884 (78.1)	2582 (5.1)	47,582 (94.9)
6	50,168 (20.8)	190,856 (79.2)	1996 (4.4)	43,845 (95.6)
7	33,992 (19.4)	141,336 (80.6)	1407 (4.4)	30,429 (95.6)
8	30,682 (17.6)	143,192 (82.4)	1284 (4.3)	28,910 (95.7)
9	24,273 (22.2)	85,210 (77.8)	1023 (5.5)	17,460 (94.5)
10 (least deprived)	12,947 (29.4)	31,024 (70.6)	459 (6.9)	6151 (93.1)
Missing	519 (13.2)	3399 (86.8)	18 (2.6)	684 (97.4)
Care home resident	1366 (42.0)	1887 (58.0)	69 (4.0)	1664 (96.0)

a Observed positive and negative categories refer only to patients with an ascertained observed hypertension status; percentages are calculated using this subgroup as the denominator.

b IMD: Index of Multiple Deprivation.

The area under the receiver operating characteristic curve (AUROC) for the model’s predictions was 0.67. At the 60% threshold, the model’s sensitivity was 62.7% (95% CI 62.5-62.8), and the specificity was 60.7% (95% CI 60.5-60.8; Table 4). Based on national estimates of hypertension, prevalence ranged from 22.4% to 32% [1,3,27]. The corresponding positive predictive values (PPVs) ranged from 31.5% (95% CI 31.2-31.8) to 42.9% (95% CI 42.5-43.2), and the NPVs ranged from 77.6% (95% CI 77.2-77.9) to 84.9% (95% CI 84.7-85.2; Table 5).

**Table 4.** Confusion matrix for model predictions against observed hypertension status, overall sensitivity and specificity of the model.

Hypertension status	Predicted positive, n/N (%)	Predicted negative, n/N (%)
Hypertension	10,065/16,061 (62.7)	5996/16,061 (37.3)
No hypertension	127,840/325,177 (39.3)	197,337/325,177 (60.7)

**Table 5.** Positive predictive values (PPVs) and negative predictive values (NPVs) based on reported hypertension prevalence estimates in England (2017) and North West London (2015-2019).

Source of prevalence estimate	PPV, % (95% CI)	NPV, % (95% CI)
Office of National Statistics^a^	42.9 (42.5-43.2)	77.6 (77.2-77.9)
National Institute for Health and Care Excellence^b^	36.1 (35.8-36.4)	82.1 (81.8-82.4)
Public Health England^c^	31.5 (31.2-31.8)	84.9 (84.7-85.2)

a 32% of adults in England, according to the Office of National Statistics [1].

b 26.2% of adults in England in 2017, according to the National Institute for Health and Care Excellence [3].

c 22.4% of adults in North West London Sustainability and Transformation Partnership, according to a 2017 Public Health England report [27].

The sensitivity of the model’s predictions was highest in the oldest age group (65 years or older, odds ratio [OR] 294.0, 95% CI 244.2-355.5) compared with the reference group aged less than 49 years, in Black patients (OR 1.72, 95% CI 1.45-2.04) and among those in the most deprived quintile (reference group; Table 6). Sensitivity decreased with decreasing socioeconomic deprivation, until plateauing at a comparable level in the second-least and least deprived quintiles. The extremely high OR seen for the older age groups here were consistent with the high proportions predicted to be positive for these groups (Table 3), since the odds increase indefinitely as this proportion approaches 100%. Sensitivity was comparable between men and women (male OR 0.95, 95% CI 0.87-1.04).

Specificity of the model’s predictions was highest in the youngest age group (<49 y, reference group), females (reference group), White patients (OR 8.11, 95% CI 7.86-8.37), and the second-least deprived quintile (OR 4.76, 95% CI 4.57-4.96). Conversely to the pattern for sensitivity, specificity decreased with age and increased with decreasing socioeconomic deprivation, until plateauing in the second-least and least deprived quintiles. Predictions for those of other ethnic groups (OR 5.57, 95% CI 5.34-5.81) and mixed ethnicity (OR 5.29, 95% CI 4.94-5.66) had similarly high specificity to the White ethnic group, albeit slightly lower. Predictions for Black patients had the lowest specificity (OR 0.67, 95% CI 0.64-0.69; Table 6).

**Table 6.** Logistic regression models for sensitivity and specificity of model predictions in the study population^a^: patients registered with a North West London general practice, aged 16 or older, with no prior diagnosis of hypertension as of May 19, 2023.

Characteristic	Outcome: odds of receiving a positive prediction (sensitivity)		Outcome: odds of receiving a negative prediction (specificity)	
	OR^b^ (95% CI)	*P* value	OR (95% CI)	*P* value
Reference categories^c^ (<49 y, female, Asian or Asian British, IMD^d^ 1)	0.65 (0.55-0.76)	<.001	3.01 (2.91-3.12)	<.001
Age (y)		<.001		<.001
<49	—^e^		—	
50‐64	31.8 (28.1-36.1)		0.019 (0.019-0.019)	
≥65	294.0 (244.2-355.5)		0.002 (0.002-0.002)	
Gender				
Female	—		—	
Male	0.95 (0.87-1.04)	.26	0.92 (0.90-0.94)	<.001
Ethnicity		<.001		<.001
Asian or Asian British	—		—	
Black or Black British	1.72 (1.45-2.04)		0.67 (0.64-0.69)	
Other ethnic groups	0.16 (0.13-0.19)		5.57 (5.34-5.81)	
Mixed	0.21 (0.16-0.28)		5.29 (4.94-5.66)	
White	0.15 (0.13-0.17)		8.11 (7.86-8.37)	
IMD quintile		<.001		<.001
1 (most deprived)	—		—	
2	0.68 (0.59-0.79)		1.53 (1.48-1.58)	
3	0.46 (0.39-0.54)		2.27 (2.19-2.36)	
4	0.30 (0.25-0.36)		4.76 (4.57-4.96)	
5 (least deprived)	0.35 (0.29-0.42)		4.58 (4.36-4.82)	

a Patients with missing data on ethnicity or deprivation were excluded from these models thus restricting the sample size to N=337,552.

b OR: odds ratio.

c Odds, rather than odds ratios, are shown for the model intercept (reference category for all variables).

d IMD: Index of Multiple Deprivation.

e Not applicable.

Predictions made for the ascertained population using a logistic regression model including age group, gender, ethnicity, and IMD quintile and trained on an up-sampled dataset yielded an AUROC of 0.66, and selecting an optimal threshold of 50.6% for this model gave a sensitivity of 62.8% and specificity of 61.0%. The same approach, using only age group as a predictor, yielded an AUROC of 0.62, with sensitivity of 69.4% and specificity of 54.0% at an optimal threshold of 47.7% (Table S11 in Multimedia Appendix 1).

The sensitivity analysis excluding those who died or deregistered from their GP during the study period yielded very similar results for overall sensitivity (62.8%) and specificity (60.4%; Table S2 in Multimedia Appendix 1). OR for demographics associated with sensitivity and specificity were also very similar to the main analysis (Tables S3 and S4 in Multimedia Appendix 1). We also conducted a sensitivity analysis classifying those who did not have a positive hypertension diagnosis as not having hypertension, to establish whether and how our results may be affected by any systematic differences between those with an ascertained hypertension status and those without. The results for model sensitivity were identical, whereas the overall specificity increased by 17 percentage points (Tables S5 and S8 in Multimedia Appendix 1). Specificity of the model’s predictions was highest in the youngest age group (<49 y reference group), males (OR 1.08, 95% CI 1.07-1.10), White patients (OR 6.85, 95% CI 6.74-6.96), and the second-least deprived quintile (OR 4.56, 95% CI 4.47-4.66; Table S6 in Multimedia Appendix 1).

For a more thorough understanding of how sensitivity and specificity varied across possible combinations of demographics, we ranked combinations of demographics with the 20 highest and lowest sensitivity and specificity values. Sensitivity was highest in Asian or Asian British males aged over 65 years living in the most deprived quintile, and specificity was highest in White females aged under 49 years living in the second-least deprived quintile (Tables S9 and S10 in Multimedia Appendix 1).

## Discussion

### Summary of Principal Results

We conducted an independent pragmatic evaluation of a third-party predictive model, commissioned by NWL integrated care board, using new data not used in training and validation of the model. The model was able to correctly identify 62.7% (10,065/16,061) of cases of undiagnosed hypertension and 60.7% (197,337/325,177) of people without hypertension. Both the sensitivity and specificity of the model were moderate, which means that there will be a large number of missed cases as well as false positives, leading to some people not receiving the help they need and others being called in for a BP check when not necessary, potentially wasting resources.

We also found that the model’s performance varied across demographic groups. The results showed that the model was significantly better at ruling out hypertension in White patients (OR 8.11, 95% CI 7.86-8.37) compared with Asian patients, as demonstrated by the large disparity in specificity between the 2 groups. Furthermore, though sensitivity was high in Black patients (OR 1.72, 95% CI 1.45-2.04), specificity was lowest in this group (OR 0.67, 95% CI 0.64-0.69), which indicates a disproportionate rate of false positives for these patients. The results showing the differences in model performance between demographic groups should be handled with caution, as participants in groups with higher rates of false-positive predictions also tend to have higher rates of misdiagnosis [28].

We found that a simple approach using logistic regression based on demographic variables achieved comparable performance to the random forest model, in terms of AUROC (0.66 vs 0.67, respectively), sensitivity (62.8% vs 62.7%), and specificity (61.0% vs 60.7%). A regression model using only age group achieved higher sensitivity at the cost of lower specificity. Overall, these findings indicate that simple regression models should be considered alongside more complicated machine-learning models. When the transparency afforded by the straightforward interpretation of regression model coefficients is not outweighed by significant increases in predictive power, more transparent models should be preferred.

The results of our sensitivity analysis indicated that, with respect to most variables, our findings were not affected by systematic differences between those who were ascertained during the study period and those who were not. However, there was a difference in the effect of gender on the specificity of the model, indicating that there may be systematic differences in relation to gender between those who were ascertained during the study period and those who were not. The fact that women were more likely to be ascertained than men, in combination with the fact that most people did not get diagnosed with hypertension during the study period, meant that the main analysis excludes more true negatives for men than for women. This is why the specificity is higher in men when including those who were not ascertained as negatives, whereas it is higher in women when using only confirmed negatives in the main analysis. However, given that a proportion of the nonascertained group will, in fact, have hypertension, the finding of higher specificity in men in the sensitivity analysis should be interpreted with caution. The finding that women were more likely to be ascertained than men is consistent with the literature indicating that men are less likely than women to have consulted a GP in the last 12 months and have fewer health care consultations than women after accounting for consultations related to reproductive issues [29-31].

Using national estimates of true prevalence, the PPV, the proportion of correct positive predictions made by the model, ranged from 31.5% (95% CI 31.2-31.8) to 42.9% (95% CI 42.5-43.2). Estimates of the NPV, the proportion of correct negative predictions made by the model, ranged from 77.6% (95% CI 77.2-77.9) to 84.9% (95% CI 84.7-85.2). Overall, the PPV was low, indicating that the model was not reliable in its positive predictions and that there was a high likelihood of false positives. However, the NPV was high, demonstrating that false negatives were less likely and negative predictions were more reliable.

### Findings in the Context of Existing Literature

Compared with previous studies that used large-scale EHR data for hypertension risk prediction and case-finding, this study is uniquely positioned to provide a real-world evaluation of model performance across demographic features and the implications for its use in practice [13-16]. Our study was an independent evaluation of a sector-commissioned case-finding model, whereas other studies primarily involved the development and in-house validation of these models. For instance, 1 study developed a model predicting the 2-year risk of hypertension and prospectively validated it in a separate cohort. This model was found to predict hypertension effectively; however, this study only included patients in the desert regions of northwest China, reducing the generalizability of the results to larger, more diverse populations. However, consistent with our study, which found age to be by far the most influential variable in the model, Cheng et al [14] found age to be a significant predictor of the risk of incident hypertension over 2 years.

Another study compared several different algorithms in the development of a hypertension risk prediction model and validated the model in 2 countries, using a Korean training cohort and a Japanese testing cohort [15]. This study also compared the 5-fold cross-validation accuracy across 6 machine-learning models and found that the random forest model performed well in terms of specificity and overall accuracy but had low sensitivity, consistent with the performance of the commissioned model we evaluated [15].

A 2022 systematic review of hypertension prediction machine-learning models found that there is an increasing trend in models to predict hypertension with moderate to high predictive performance. These models used a variety of methods and included a range of features, such as clinical, socioeconomic, and nutritional predictors of hypertension [16].

A 2018 systematic review, which summarized evidence on interventions to improve detection of hypertension in primary care, found that ethnic and socioeconomic differences were not major influencing factors in detection among older age groups, whereas social isolation was an influential factor [32]. Our results, however, showed that the model performed better at identifying cases in older, Black, and more deprived groups, while it was better at ruling out hypertension in younger, White, and less deprived groups. These results are positive with respect to the fact that 1 focus of the national CORE20PLUS5 approach in England is hypertension case-finding in the most deprived 20% of the population [33].

A 2017 systematic review analyzed 107 studies on risk prediction models that used EHR data. These models were developed to infer the clinical status of patients where this was unknown [12]. This review called for improvements in the use of evaluation metrics, such as PPV, which take prevalence into account, to better assess the clinical utility of the developed models. Our study evaluated the PPV and NPV using national estimates of hypertension prevalence to get a range of values that could indicate the reliability of the model’s predictions in a more realistic and clinically useful degree.

This illustrates the broader need for high-quality, independent evaluation of commissioned statistical and machine-learning models, especially where such models are intended for use in clinical care or management. With health systems internationally operating within tightly constrained resources, it is important that analytic solutions using routinely collected data have real-world value and consider health equity.

### Strengths and Limitations

A strength of our evaluation was that it was an independent, retrospective, real-world, postdeployment evaluation of a predictive model for undiagnosed hypertension in a large population. We used confirmed diagnoses to ascertain positive cases of hypertension rather than proxy measures such as above-threshold BP measurements—an approach that strengthens clinical relevance and bridges the gap between prediction and real-world utility. This evaluation occurred before the results of the model were implemented, ensuring that the study is not measuring the intervention’s yield rather than the independent predictive performance of the case-finding model. Using information about clinical hypertension diagnosis and measurements from GP records combines 2 indicators of hypertension status. This is also a key strength compared to methods common in the literature, which rely only on BP measurements and thresholds to classify true positive and negative cases, or use information about a formal diagnosis and assume that the absence of a diagnosis is equivalent to confirmation of negative hypertension status. This is especially problematic given the premise of significant numbers of undiagnosed cases in the population. The results were robust to excluding those who died or left their GP during the study period, and only the effect of gender changed when including patients whose hypertension status was not ascertained during the study.

A novel aspect of our evaluation is its focus on equity in model performance, which is often overlooked in evaluation studies [20]. This study used the WSIC dataset, which provides high coverage of the demographically diverse NWL population [18]. By investigating the predictive performance of the model across social determinants of health using the WSIC data, our evaluation study aligns with a growing recognition of fairness as a foundational criterion in the assessment of clinical models, not an afterthought [21]. Such equity-focused evaluation remains relatively rare in deployed models, especially in the context of detecting undiagnosed conditions [13-15,22].

A limitation of our approach is that it depends on the accuracy of the coding. We assumed that GPs had followed up with all patients with elevated BP measurements and that there had been no long delay in diagnosing hypertension. Although this is within the framework of clinical decision-making, there may be patients who were not diagnosed with hypertension because of a lack of follow-up or nonadherence to guidelines by the GPs. This would result in some patients being wrongly classified as not having hypertension when, in fact, they did have the condition. We also did not account for time to diagnosis, loss to follow-up, or the competing risk of death. However, our sensitivity analysis, excluding those who died or left the practice for another outside NWL, mitigated this limitation to some extent. Lastly, the scope of this study did not include examination of design decisions made in the model development phase, or issues such as feature importance. Future studies evaluating predictive models should consider including such analyses to provide insights into how the model is working, in addition to how well it is working.

### Implications

Overall, this evaluation contributes to the emerging field of public health data science by demonstrating how independent evaluation within the same health system can reveal demographic biases and performance variations that may not be apparent during model development. Use of the predictive model has the potential to identify a considerable portion of people living with undiagnosed hypertension, with a view to providing earlier treatment and better health outcomes, although more transparent models are likely to offer similar benefits. Current practice in the NHS is for every individual without preexisting cardiovascular disease, diabetes, or kidney disease to be offered health checks every 5 years from the age of 40 years to 74 years [34]. Use of the model could therefore enable diagnoses earlier in the time periods between health checks. However, a potentially more important group from the perspective of case-finding is younger people who are not offered health checks, and the model demonstrated negligible utility among these groups.

A limitation of the model was the use of area-level proxies in the census data to infer individual clinical risk [35]. Although the census data used was at the most granular level, the ecological fallacy could be a concern when interpreting the model’s predictions. The model was also limited by the available data, including missingness of information in variables including ethnicity, deprivation, language, and religion. As a result, the model may have, to some extent, captured variation in documentation rather than underlying clinical associations.

While the model offers moderate sensitivity and specificity, it is still more likely that a prediction of hypertension is wrong than right. Furthermore, there were wide variations in sensitivity and specificity by demographic groups. Therefore, while the model may be useful as part of a targeted screening program to identify patients likely to benefit from having their BP measured, it should be considered alongside other strategies to ensure certain groups are not disadvantaged, such as enhanced community BP screening and opportunistic measurement during health care visits. For example, given that the model performs poorly in identifying undiagnosed hypertension among younger people, there may be a need for a separate strategy focused on this group.

When integrating the model alongside other strategies, one approach that could be of use would be to deploy the model within strata of the population where it performs well and use alternative strategies where it performs less well. For instance, tables showing descending cumulative sensitivity and specificity are shown in Multimedia Appendix 1 and could be used to identify strata where the model may be of use (Tables S9 and S10 in Multimedia Appendix 1). Alternatively, the model could be deployed across the whole population, with additional strategies supplementing the predictions made for population strata where the model performs poorly.

Several other considerations could be made for the continued and improved use of this model. First, conducting studies to routinely evaluate the model’s predictions over a longer period and the impact of the use of model results on clinical outcomes could greatly improve the understanding of its impact and utility as a practical tool for population health. Second, the findings of this analysis could be incorporated into future cost-benefit analyses to explore the financial impact of such case-finding models on the health system. Finally, collaborating with policymakers to use the findings to inform health care policies and resource allocation, based on analyses like ours and especially by targeting high-risk groups for hypertension screening, could improve the detection and treatment of undiagnosed hypertension, with consequent improvements in health outcomes in NWL and beyond.

### Future Research

The results of this analysis are well-placed to inform deployment of the model alongside other strategies in practice, to drive further enhancements to the model, guide future cost-benefit analyses, steer resource allocation for the detection of undiagnosed hypertension, and contribute to health equity efforts and inform public health policy in NWL. Future research into machine learning techniques that incorporate equity more intentionally is needed to improve the development of such case-finding tools for hypertension. Future work could also include a randomized prospective study to minimize potential misclassification bias due to incomplete patient follow-up or variation in GPs’ application of diagnosis guidelines.

### Conclusion

This independent pragmatic evaluation highlights both the value and limitations of predictive modeling for addressing undiagnosed hypertension—a major and persistent public health challenge. The findings of this study show that the model’s predictions for those without hypertension were more reliable than its predictions for those with the condition. The sensitivity and specificity of the model varied across age, ethnicity, and socioeconomic deprivation. Few predictive models in practice are evaluated for bias, real-world performance, or against more parsimonious models, and our findings illustrate why such assessments are critical for equitable and effective implementation. These findings highlight the model’s potential utility in targeting groups for hypertension screening rather than for clinical use with individual patients, emphasizing the need for tailored strategies across different demographic groups to enhance early detection and management of hypertension with a focus on health equity.

## Supplementary material

10.2196/87084 Multimedia Appendix 1 Additional analyses and results.

## Abbreviations

ARC: Applied Research Collaboration
AUROC: area under the receiver operating characteristic
BP: blood pressure
EHR: electronic health record
GP: general practice
IMD: Index of Multiple Deprivation
NHS: National Health Service
NICE: National Institute for Health and Care Excellence
NIHR: National Institute for Health Research
NPV: negative predictive value
NWL: North West London
ONS: Office of National Statistics
OR: odds ratio
PHE: Public Health England
PPV: positive predictive value
QOF: Quality and Outcomes Framework
STP: Sustainability and Transformation Partnership
WSIC: Whole Systems Integrated Care
