# Supplementary material for: Evaluation of a National Health Service Machine-Learning Model for Hypertension Case-Finding: Retrospective Cohort Study
Source: J Med Internet Res. 2026 Sep 15;28:e87084. doi: 10.2196/87084 (PMC13577441; doi:10.2196/87084)
Supplement: Supplementary file 1 [file jmir-v28-e87084-s001.docx]

# Supplementary materials

# Features included in the model

Supplementary Results

Table S1: Confusion matrix with overall sensitivity and specificity for 50% probability threshold

|  | **Predicted Positive** | **Predicted Negative** | **Total** |  |
| --- | --- | --- | --- | --- |
| **Hypertension** | 12,578 (78.3%) | 3,483 (21.7%) | *16,061* | *Sensitivity:* 78.3% |
| **No Hypertension** | 172,185 (53.0%) | 152,992 (47.0%) | *325,177* | *Specificity:* 47.0% |

### Sensitivity analyses

#### Sensitivity analysis 1: Exclusion of those who died or deregistered from their GP during the study period

Table S2: Confusion matrix with overall sensitivity and specificity for 60% probability threshold

|  | **Predicted Positive** | **Predicted Negative** | **Total** |  |
| --- | --- | --- | --- | --- |
| **Hypertension** | 9,927 (62.8%) | 5,882 (37.2%) | *15,809* | *Sensitivity:* 62.8% |
| **No Hypertension** | 125,104 (39.6%) | 190,769 (60.4%) | *315,873* | *Specificity:* 60.4% |

Table S3: Logistic regression model for sensitivity

| Characteristic | OR | 95% CI | p-value |
| --- | --- | --- | --- |
| Reference categories* (<49, Female, Asian or Asian British, IMD 1) | 0.65 | (0.56, 0.77) | <0.001 |
| Age (years) |  |  |  |
| <49 | — | — |  |
| 50-64 | 31.4 | (27.7, 35.7) | <0.001 |
| 65+ | 293.5 | (243.4, 355.6) | <0.001 |
| Gender |  |  |  |
| Female | — | — |  |
| Male | 0.95 | (0.86, 1.04) | 0.23 |
| Ethnicity |  |  |  |
| Asian or Asian British | — | — |  |
| Black or Black British | 1.72 | (1.45, 2.04) | <0.001 |
| Other ethnic groups | 0.16 | (0.13, 0.19) | <0.001 |
| Mixed | 0.22 | (0.16, 0.29) | <0.001 |
| White | 0.15 | (0.13, 0.17) | <0.001 |
| IMD quintile |  |  |  |
| 1 (Most deprived) | — | — |  |
| 2 | 0.69 | (0.59, 0.80) | <0.001 |
| 3 | 0.46 | (0.40, 0.54) | <0.001 |
| 4 | 0.30 | (0.25, 0.36) | <0.001 |
| 5 (Least deprived) | 0.35 | (0.29, 0.43) | <0.001 |

*Odds

Table S4: Logistic regression model for specificity

| Characteristic | OR | 95% CI | p-value |
| --- | --- | --- | --- |
| Reference categories* (<49, Female, Asian or Asian British, IMD 1) | 2.96 | (2.86, 3.07) | <0.001 |
| Age (years) |  |  |  |
| <49 | — | — |  |
| 50-64 | 0.019 | (0.018, 0.019) | <0.001 |
| 65+ | 0.002 | (0.002, 0.002) | <0.001 |
| Gender |  |  |  |
| Female | — | — |  |
| Male | 0.92 | (0.90, 0.95) | <0.001 |
| Ethnicity |  |  |  |
| Asian or Asian British | — | — |  |
| Black or Black British | 0.67 | (0.64, 0.70) | <0.001 |
| Other ethnic groups | 5.60 | (5.36, 5.84) | <0.001 |
| Mixed | 5.32 | (4.97, 5.70) | <0.001 |
| White | 8.15 | (7.89, 8.41) | <0.001 |
| IMD quintile |  |  |  |
| 1 (Most deprived) | — | — |  |
| 2 | 1.53 | (1.48, 1.58) | <0.001 |
| 3 | 2.27 | (2.19, 2.36) | <0.001 |
| 4 | 4.82 | (4.64, 5.03) | <0.001 |
| 5 (Least deprived) | 4.63 | (4.40, 4.88) | <0.001 |

*Odds

#### Sensitivity analysis 2: Classifying those who did not have a positive hypertension diagnosis as not having hypertension

Table S5: Logistic regression model for sensitivity

| Characteristic | OR | 95% CI | p-value |
| --- | --- | --- | --- |
| Reference categories* (<49, Female, Asian or Asian British, IMD 1) | 0.65 | (0.55, 0.76) | <0.001 |
| Age (years) |  |  |  |
| <49 | — | — |  |
| 50-64 | 31.8 | (28.1, 36.1) | <0.001 |
| 65+ | 294.0 | (244.2, 355.5) | <0.001 |
| Gender |  |  |  |
| Female | — | — |  |
| Male | 0.95 | (0.87, 1.04) | 0.26 |
| Ethnicity |  |  |  |
| Asian or Asian British | — | — |  |
| Black or Black British | 1.72 | (1.45, 2.04) | <0.001 |
| Other ethnic groups | 0.16 | (0.13, 0.19) | <0.001 |
| Mixed | 0.21 | (0.16, 0.28) | <0.001 |
| White | 0.15 | (0.13, 0.17) | <0.001 |
| IMD quintile |  |  |  |
| 1 (Most deprived) | — | — |  |
| 2 | 0.68 | (0.59, 0.79) | <0.001 |
| 3 | 0.46 | (0.39, 0.54) | <0.001 |
| 4 | 0.30 | (0.25, 0.36) | <0.001 |
| 5 (Least deprived) | 0.35 | (0.29, 0.42) | <0.001 |

*Odds

Table S6: Logistic regression model for specificity

| Characteristic | OR | 95% CI | p-value |
| --- | --- | --- | --- |
| Reference categories* (<49, Female, Asian or Asian British, IMD 1) | 4.55 | (4.47, 4.64) | <0.001 |
| Age (years) |  |  |  |
| <49 | — | — |  |
| 50-64 | 0.013 | (0.013, 0.013) | <0.001 |
| 65+ | 0.0013 | (0.0012, 0.0013) | <0.001 |
| Gender |  |  |  |
| Female | — | — |  |
| Male | 1.08 | (1.07, 1.10) | <0.001 |
| Ethnicity |  |  |  |
| Asian or Asian British | — | — |  |
| Black or Black British | 0.63 | (0.62, 0.64) | <0.001 |
| Other ethnic groups | 5.96 | (5.83, 6.09) | <0.001 |
| Mixed | 4.98 | (4.80, 5.17) | <0.001 |
| White | 6.85 | (6.74, 6.96) | <0.001 |
| IMD quintile |  |  |  |
| 1 (Most deprived) | — | — |  |
| 2 | 1.69 | (1.66, 1.72) | <0.001 |
| 3 | 2.39 | (2.35, 2.44) | <0.001 |
| 4 | 4.56 | (4.47, 4.66) | <0.001 |
| 5 (Least deprived) | 3.24 | (3.16, 3.32) | <0.001 |

*Odds

Table S7: Confusion matrix with overall sensitivity and specificity for 50% probability threshold

|  | **Predicted Positive** | **Predicted Negative** | **Total** |  |
| --- | --- | --- | --- | --- |
| **Hypertension** | 12,578 (78.3%) | 3,483 (21.7%) | *16,061* | *Sensitivity:* 78.3% |
| **No Hypertension** | 576,077 (32.3%) | 1,207,863 (67.7%) | *1,783,940* | *Specificity:* 67.7% |

Table S8: Confusion matrix with overall sensitivity and specificity for 60% probability threshold

|  | **Predicted Positive** | **Predicted Negative** | **Total** |  |
| --- | --- | --- | --- | --- |
| **Hypertension** | 10,065 (62.7%) | 5,996 (37.3%) | *16,061* | *Sensitivity:* 62.7% |
| **No Hypertension** | 392,233 (22.0%) | 1,391,707 (78.0%) | *1,783,940* | *Specificity:* 78.0% |

### Ranked sensitivity and specificity tables

Table S9: Top 20 cumulative sensitivity in combinations of demographic groups

| Age group | Gender | IMD quintile | Ethnicity | Cumulative sensitivity |
| --- | --- | --- | --- | --- |
| 65+ | Male | 1 | Asian or asian british | 0.9945241 |
| 65+ | Male | 3 | Black or black british | 0.9935914 |
| 65+ | Female | 2 | Asian or asian british | 0.9931126 |
| 65+ | Female | 3 | Asian or asian british | 0.9918826 |
| 65+ | Male | 3 | Asian or asian british | 0.9910690 |
| 65+ | Male | 4 | Asian or asian british | 0.9901621 |
| 65+ | Female | 1 | White | 0.9880345 |
| 65+ | Female | 2 | White | 0.9803172 |
| 65+ | Male | 2 | Other ethnic groups | 0.9783925 |
| 65+ | Male | 2 | White | 0.9765933 |
| 50-64 | Male | 3 | Black or black british | 0.9744314 |
| 65+ | Female | 3 | White | 0.9720032 |
| 50-64 | Male | 2 | Asian or asian british | 0.9678589 |
| 65+ | Male | 3 | White | 0.9625710 |
| 65+ | Female | 4 | Mixed | 0.9609916 |
| 65+ | Female | 5 | White | 0.9589587 |
| 65+ | Male | 5 | White | 0.9550250 |
| 50-64 | Male | 3 | Asian or asian british | 0.9530859 |
| 65+ | Female | 4 | White | 0.9511619 |
| 65+ | Male | 4 | White | 0.9492041 |

Table S10: Top 20 cumulative specificity in combinations of demographic groups

| Age group | Gender | IMD quintile | Ethnicity | Cumulative specificity |
| --- | --- | --- | --- | --- |
| <49 | Female | 4 | White | 0.9914661 |
| <49 | Female | 5 | White | 0.9913756 |
| <49 | Male | 4 | White | 0.9912280 |
| <49 | Male | 5 | White | 0.9911599 |
| <49 | Female | 4 | Other ethnic groups | 0.9907797 |
| <49 | Female | 5 | Other ethnic groups | 0.9906610 |
| <49 | Female | 4 | Mixed | 0.9905403 |
| <49 | Male | 4 | Other ethnic groups | 0.9903603 |
| <49 | Female | 5 | Mixed | 0.9903206 |
| <49 | Male | 5 | Other ethnic groups | 0.9902625 |
| <49 | Male | 4 | Mixed | 0.9902082 |
| <49 | Male | 5 | Mixed | 0.9901867 |
| <49 | Female | 3 | White | 0.9875887 |
| <49 | Male | 3 | White | 0.9866993 |
| <49 | Female | 3 | Other ethnic groups | 0.9858165 |
| <49 | Female | 2 | White | 0.9833010 |
| <49 | Female | 3 | Mixed | 0.9831144 |
| <49 | Male | 3 | Other ethnic groups | 0.9827935 |
| <49 | Male | 2 | White | 0.9818470 |
| <49 | Male | 3 | Mixed | 0.9817594 |

# Comparator regression models

Table S11: Results of comparator predictive regression models on the ascertained population. Models were trained on a) up-sampled data retaining all instances of no hypertension and sampling instances of hypertension with replacement b) down-sampled data retaining all instances of hypertension and sampling a subset of instances of no hypertension, each achieving a balanced training dataset. Two logistic regression models were trained on each of these training datasets, i) including all demographic variables as predictors (age band, gender, ethnicity, IMD quintile) ii) including only age band as the predictor. n_u_, n_d_ are the number of observations in each training dataset; p_t_ is the optimal threshold defining binary predictions from predicted probabilities. Accuracy, sensitivity (sens.) and specificity (spec.) are given for each model, along with the confusion matrix. Optimal thresholds were established for each model as the point on the receiver-operator curve closest to the “perfect” model with specificity and sensitivity 100%, this approach yielded higher accuracy than using Youden’s index in this case.

|  |  |  | Hypertension status  *Predictions for all ascertained participants n = 341,238* | |
| --- | --- | --- | --- | --- |
| Training Dataset | Variables Included |  | Hypertension | No Hypertension |
| (a) Up-sampled (n_u_ = 650,354) | (i) All demographics  (AUROC = 0.66, p_t_ = 50.6%) | Predicted positive | 10,083 | 126,665 |
|  |  | Predicted negative | 5,978 | 198,512 |
|  |  | Accuracy: 61.1% | Sens.: 62.8% | Spec.: 61.0% |
|  |  |  |  |  |
|  | (ii) Age only*  (AUROC = 0.62,  p_t_ = 47.7%) | Predicted positive | 11,145 | 149,433 |
|  |  | Predicted negative | 4,916 | 175,744 |
|  |  | Accuracy: 54.8% | Sens.: 69.4% | Spec.: 54.0% |
|  |  |  |  |  |
| (b) Down-sampled  (n_d_ = 32,122) | (i) All demographics  (AUROC = 0.66,  p_t_ = 49.4%) | Predicted positive | 10,635 | 137,748 |
|  |  | Predicted negative | 5,426 | 187,429 |
|  |  | Accuracy: 58.0% | Sens.: 66.2% | Spec.: 57.6% |
|  |  |  |  |  |
|  | (ii) Age only*  (AUROC = 0.62,  p_t_ = 47.5%) | Predicted positive | 11,145 | 149,433 |
|  |  | Predicted negative | 4,916 | 175,744 |
|  |  | Accuracy: 54.8% | Sens.: 69.4% | Spec.: 54.0% |

** Note that the two age-only models produce identical confusion matrices, despite being trained on different samples of the data, and having slightly different optimal thresholds. A single categorical predictor with only three levels yields only three distinct predicted probabilities. The two thresholds selected here for the age-only models fall between the same pair of predicted values and hence result in identical binary classifications.*

The random forest model was assessed at developer-selected threshold probability. We chose to use optimal thresholds for the comparator regression models because there is no reason to compare the random forest model with a logistic regression model at a poorly optimised threshold. Such a comparison would not reveal anything useful about the comparative accuracy of the predictions made by the two models. By comparing the random forest at the developer-selected threshold against regression models using an optimal threshold, we are able to quantify the performance of the random forest model against the best possible predictions made by the comparator models. Furthermore, the comparator models were fitted, and the thresholds optimised, on the same ascertained cohort they were evaluated against. Were the random forest to outperform even the most optimal comparator predictions, the benefit of the more complex machine learning model would be apparent.
